# A Formative Evaluation of Interventions to Enhance Clinical Trial Diversity Guided by the Socioecological Model

**DOI:** 10.3390/cancers17142282

**Published:** 2025-07-09

**Authors:** Melany Garcia, Carley Geiss, Rebecca Blackwell, Melinda L. Maconi, Rossybelle P. Amorrortu, Elliott Tapia-Kwan, Kea Turner, Lindsay Fuzzell, Yayi Zhao, Steven A. Eschrich, Dana E. Rollison, Susan T. Vadaparampil

**Affiliations:** 1 Department of Health Outcomes & Behavior, Moffitt Cancer Center, Tampa, FL 33612, USA; melany.garcia@moffitt.org (M.G.); rossybelle.amorrortu@moffitt.org (R.P.A.); lindsay.fuzzell@moffitt.org (L.F.); yayi.zhao@moffitt.org (Y.Z.); 2 Participant Research, Interventions, & Measurements Core, Moffitt Cancer Center, Tampa, FL 33612, USA; carley.geiss@moffitt.org (C.G.); rblackwell@usf.edu (R.B.); melinda.maconi@moffitt.org (M.L.M.); 3 Department of Sociology and Interdisciplinary Social Sciences, University of South Florida, Tampa, FL 33620, USA; 4 Non-Therapeutics Research Office, Moffitt Cancer Center, Tampa, FL 33612, USA; elliott.tapia-kwan@moffitt.org; 5 School of Nursing, Division of Health Systems, Policy, and Innovation, University of North Carolina at Chapel Hill, Chapel Hill, NC 27514, USA; kea.turner@unc.edu; 6 Department of Biostatistics and Bioinformatics, Moffitt Cancer Center, Tampa, FL 33612, USA; steven.eschrich@moffitt.org; 7 Department of Cancer Epidemiology, Moffitt Cancer Center, Tampa, FL 33612, USA; rollisondana@gmail.com; 8 Office of Community Outreach and Engagement, Moffitt Cancer Center, Tampa, FL 33612, USA

**Keywords:** cancer clinical trials, disparities, minority populations, community outreach, clinical trials education, multilevel interventions, socioecological model

## Abstract

Background/objectives: Racial and ethnic minority patients are underrepresented in cancer clinical trials (CCTs) and multilevel strategies are required to increase participation. This study describes barriers and facilitators to minority CCT participation alongside feedback on a multilevel intervention (MLI) designed to reduce participation barriers, as posited by the socioecological model (SEM). Methods: Interviews with Moffitt Cancer Center (MCC) physicians, community physicians, patients with cancer, community residents, and clinical research coordinators (CRCs) were conducted from June 2023–February 2024. Verbal responses were analyzed using thematic analysis and categorized into SEM levels. Mean helpfulness scores rating interventions (from 1 (not helpful) to 5 (very helpful)) were summarized. Results: Approximately 50 interviews were completed. Thematic findings confirmed CCT referral and enrollment barriers across all SEM levels. At the community level, MCC patients and community residents felt that community health educators can improve patient experiences and suggested they connect patients to social/financial resources, assist with patient registration, and provide CCT education. While physicians and CRCs reacted positively to all institutional-level tools, the highest scored tool simultaneously addressed CCT referral and enrollment at the institution (e.g., trial identification/referrals) and interpersonal level (communication platform for community and MCC physicians) (mean = 4.27). At the intrapersonal level, patients were enthusiastic about a digital CCT decision aid (mean = 4.53) and suggested its integration into MCC’s patient portal. Conclusions: Results underscore the value of conducting formative research to tailor interventions to target population needs. Our approach can be leveraged by future researchers seeking to evaluate MLIs addressing additional CCT challenges or broader health topics.

## Simple Summary

Racial and ethnic minority patients are underrepresented in cancer clinical trials (CCTs) due to barriers at the intrapersonal, interpersonal, institutional, and community levels. While previous studies aimed to increase minority CCT participation, none have addressed multiple barriers at once. Additionally, formative research is used to inform multilevel interventions (MLIs) aimed at reducing health disparities and is an important step to deploy interventions in real world settings. Therefore, the purpose of this article is to provide an overview of a formative research process to identify (1) barriers and facilitators to minority CCT participation and (2) perspectives on digital and community outreach interventions among community and cancer center end-user groups. This process is particularly important for future research seeking to engage end users in the design and development of targeted MLIs to reduce barriers to CCT participation and health disparities more broadly.

## 1. Introduction

Non-Hispanic (NH) Black/African American (AA) and Hispanic populations are underrepresented in cancer clinical trials (CCTs) and experience barriers to CCT referral and enrollment across multiple levels [1]. These include barriers posited within the socioecological model (SEM) [2] at the intrapersonal (e.g., patient and physician knowledge, awareness, attitudes, financial barriers) [3,4,5,6,7,8,9,10,11,12,13,14,15], interpersonal (e.g., relationships between patients and physicians) [6,8,11,12,13,14,15,16,17,18], institutional (e.g., trial availability, restrictive eligibility criteria, complex referral processes, logistical challenges) [1,8,14,15,16,17,19,20], and community levels (e.g., lack of community engagement and appropriate trials in community settings) [1,21].

Previous interventions have strived to increase minority CCT participation at the patient- and physician-level, including in a single-session educational program [22] with digital tools aimed at enhancing research literacy and CCT decision-making for minority patients [23,24,25], implicit bias training for physicians [26], and a clinical trial site self-assessment tool designed to review data on clinical trial site screening and enrollment [27]. However, these studies did not address CCT barriers across multiple levels simultaneously. Given the abundance of literature and recent frameworks suggesting that multilevel interventions (MLIs) are needed to increase patient participation in trials [18,28,29,30,31], we used an intervention development guided by the SEM needed to systematically address multilevel barriers to minority patient CCT referral and enrollment.

Formative research is being used to inform the design and development of interventions aimed at reducing health disparities [22,32,33,34,35,36]. Engaging individuals who will be targeted by an intervention (e.g., end user) [34,35,36] is an important determinant of successful MLI implementation and outcomes [37]. Quantitative and qualitative research methods have been used to elicit in-depth perspectives on barriers to CCTs among cancer patients [38,39], cancer center physicians/clinical staff [12,15,16,17,40,41], community oncology providers [12,14,15,16,18,21], and community members [41,42]. Understanding local context is also an essential step in designing interventions deployed in real-world settings [43]. Thus, we conducted interviews with end users on an MLI designed to decrease barriers to referral and enrollment of Black/AA and Hispanic patients to CCTs at the patient, physician, institutional, and community level. This report (1) identifies barriers and facilitators to CCT referral and enrollment and (2) describes end-user feedback in the context of the SEM that was used to develop and refine the MLI.

## 2. Materials and Methods

### 2.1. Overview of the ACT WONDER^2^S Study

Advancing Clinical Trials: Working Through Outreach, Navigation, and Digitally Enabled Referral and Recruitment Strategies (ACT WONDER^2^S) aims to reduce barriers to Black/AA and Hispanic patient CCT referral and enrollment through community outreach, education, and navigation efforts coupled with digital strategies for multiple target populations within the Moffitt Cancer Center (MCC) catchment area (intervention components described in Table 1). These include internal (MCC physicians, clinical research coordinators (CRCs), cancer patients) and external populations (community residents, community physicians). This study was approved by the Advarra Institutional Review Board (IRB) and the Moffitt Cancer Center Scientific Review Committee (MCC #22140).

### 2.2. Recruitment

The target sample sizes for each end-user group were guided by the principles of saturation (e.g., no new themes) in qualitative research, which is generally reached between 5 and 10 interviews per group of interest [44,45] The final sample sizes were as follows: community residents (number (n) = 5 Black/AA, n = 5 Hispanic); MCC patients (n = 5 Black/AA, n = 5 Hispanic); community physicians (n = 5 oncologists, n = 5 nononcologists); MCC CRCs (n = 10); and MCC physicians (n = 10) (n = 50 total). Recruitment strategies included flyer distribution in target clinics, social media advertisements, direct emails, and collaboration with MCC’s Patient and Family Advisory Council, advocacy, and support groups to distribute interview participation details. The MCC physician liaison team and two ACT WONDER^2^S physician coinvestigators also helped identify community physicians to advertise the interview opportunity across Tampa Bay and surrounding areas. MCC clinical departments and Clinical Trial Office leadership assisted in promoting the study to MCC physicians and CRCs. All end users provided verbal consent prior to interviews and were compensated with a USD 50 gift card via Greenphire ClinCard, a reloadable card that automates reimbursements and stipends for research participants.

### 2.3. Interviews

End users completed interviews (averaging 45 min) as part of the first of two interview phases in the ACT WONDER^2^S intervention design and development process. Interviews were conducted via phone or Zoom and were based on the SEM and simultaneously addressed (1) CCT knowledge, perceived barriers and facilitators to CCTs, current and potential outreach, referral, and enrollment strategies; and (2) feedback on how to design and refine several proposed ACT WONDER^2^S interventions before study launch. Interviews were conducted by two doctoral-level analysts (RB and CG) in the Participant Research, Interventions, and Measurement Core (PRISM) at MCC, who both have extensive training and experience in qualitative data collection. Initial drafts of the interview guides were developed by the study team, tailored to each end user group, and refined by PRISM. Structured questions were included to elicit specific information on perceived barriers and facilitators to CCTs and perspectives on the ACT WONDER^2^S intervention components. Quantitative scales (ranging from 1 (not helpful) to 5 (very helpful)) were developed by the study team to collect perceived helpfulness scores of the intervention components overall and by specific functionality to understand their utility and refine content. All questions (open- and closed-ended) were collected verbally during the interviews. 

### 2.4. Quantitative Analysis

Demographic and clinical characteristics of all end user groups were summarized by frequencies and percentages for categorical variables (e.g., sex, race, oncology specialty, etc.) and means and standard deviations (SD) for continuous variables (e.g., age [in years]). On a scale from 1 (not helpful) to 5 (very helpful), means of the scaled responses on the helpfulness of the ACT WONDER^2^S intervention components overall and by specific component features. Statistical analyses were performed via Research Electronic Data Capture (RED Cap), a web-based application software for building online surveys and databases [46].

### 2.5. Qualitative Analysis

All interviews were transcribed, deidentified, and analyzed following the tenets of applied thematic analysis [47]. Based on interview guides, emerging themes discovered during data collection, and study team meetings, an initial codebook was created and refined through the intercoder reliability process by study team members (RB and MM). Discrepancies were resolved by consensus. An acceptable level of intercoder reliability was reached (Cohen’s Kappa = 0.82) after several rounds of coding [48]. Data were then coded using NVivo 12 Plus software and synthesized into themes and subthemes by RB, MM, and CG. Subsequently, findings were categorized into levels of the SEM framework (MG).

## 3. Results

A total of 50 interviews were completed with MCC physicians (n = 10), CRCs (n = 10), MCC patients (n = 10), community physicians (n = 10), and community residents (n = 10) (Table 2). Most end-user groups were equally compromised by sex (female (50%) and male (50%)), except for MCC physicians (30% female, 70% male). Besides community physicians, less than 40% of end users were white, and most identified as non-Hispanic. The mean years of employment at MCC was 8.8 for MCC physicians (SD + 5.79, range = 3–23) and 1.4 for CRCs (SD + 0.54, range = 1–2.5) (Appendix A and Appendix B). Most MCC physicians specialized in medical oncology (90%) and 50% classified themselves as MCC physician educators who lead training and education for early-career healthcare professionals. Among community physicians, 30% reported that their healthcare system offered clinical trials and 40% had led a therapeutic CCT in the last five years. However, 80% of community physicians referred patients to MCC for cancer care and 60% referred patients to a CCT outside of their healthcare system (Appendix C).

### 3.1. Perceived Barriers, Facilitators, and Needs for Minority CCT Referral and Enrollment

**Figure 1:**
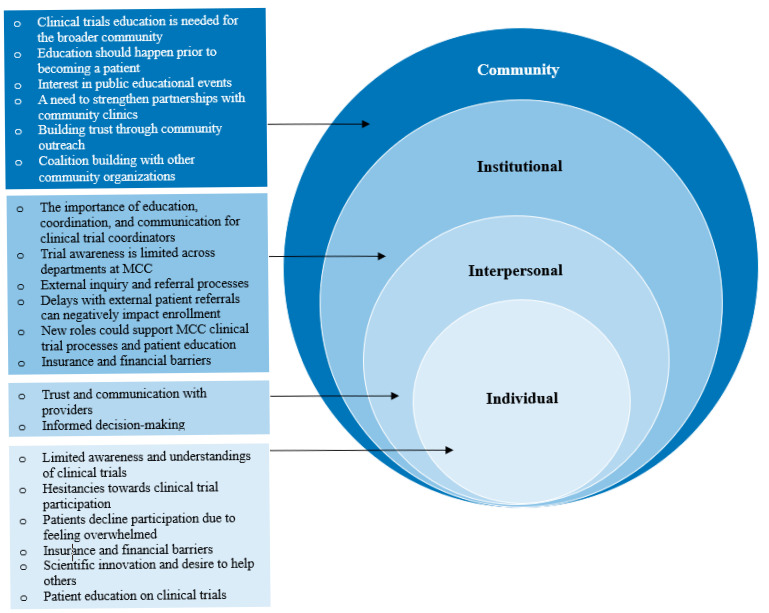
Themes grouped by levels of the socioecological model (SEM).

Themes emerged across all interviews and were organized by levels of the SEM as described below (Figure 1). Exemplary illustrative quotes are provided in Table 3.

#### 3.1.1. Community Factors

Clinical trial education for the broader community was considered essential for increasing enrollment, with some end users emphasizing the need for CCT education prior to diagnosis. MCC patients and community residents expressed interest in attending educational events held by MCC, with some reporting positive experiences attending prior events. Building trust in the community was also described as important to foster diversity in CCTs; one community resident expressed that Black and Hispanic patients often lack trust in the medical system due to historical discrimination and persistent inequalities. End users emphasized strengthening partnerships with community clinics and suggested offering continuing medical education credits and utilizing patient navigators, health educators, community liaisons, or clinical trial champions for continued outreach. Another suggestion included establishing partnerships with other institutions or entities (e.g., American Cancer Society, local government agencies, community centers, churches, and universities) to reach those who may not know about MCC or CCTs.

#### 3.1.2. Institutional Factors

While ongoing institutional coordination, communication, and education were identified as facilitators, perceived obstacles to CCT enrollment included high turnover for CRCs, complex scheduling processes, delays in receiving patient referrals/inconsistent referral patterns across institutions, and medical record sharing. Due to time constraints, MCC physicians recommended that patient navigators or health educators should educate patients on CCTs. MCC physicians and CRCs expressed limited awareness of CCTs and resources outside of their own departments, and some described relying on personal connections, research collaborations, and attendance at institutional department meetings and tumor boards for CCT information. Community physicians also reported limited awareness of availability and enrollment processes for MCC CCTs. While physicians and CRCs expressed that direct communication (e.g., email) was the most effective method of sharing information across departments and institutions, they desired an easier way to share details about upcoming CCTs due to difficulties ascertaining CCT information aside from personal connections (e.g., websites). End users also described insurance and financial factors as barriers to care, and community physicians expressed frustrations with referring patients to CCTs only to learn the patient’s insurance was not accepted by MCC.

#### 3.1.3. Interpersonal Factors

Interpersonal factors emerged as themes across MCC patients and community residents. Both groups described that collaborating with a trusted provider would enhance their confidence in making informed decisions about CCT participation. They expressed needing information from their providers on clinical trial benefits, risks, safety, side effects, drug interactions, symptom management, findings from previous phases, effectiveness, and how quickly to expect results. Conversely, MCC physicians and CRCs also described the importance of teamwork and collaborating with principal investigators within their departments to effectively prioritize patients for trial candidate selection based on disease-related factors.

#### 3.1.4. Intrapersonal Factors

Limited understanding and awareness of CCTs were noted by MCC patients, community residents, and community physicians. For instance, MCC patients and community residents were uninformed about the differences between clinical trials and standard treatment options, and community residents had less knowledge overall than MCC patients. They also reported hesitancies toward CCT participation related to potential risks (e.g., side effects, ineffectiveness), lack of trust in pharmaceutical companies/CCTs generally, discomfort in sharing health data, fear of disease progression, and feeling overwhelmed with diagnosis and treatment. Patient and physician education was described as necessary to address CCT misunderstandings, with some community physicians expressing that they were not always informed about available CCTs and associated processes. One MCC patient stated the importance of understanding the information they receive and emphasized simple, accessible language. Another patient suggested receiving CCT information through educational materials and having their questions answered during clinic visits.

### 3.2. ACT WONDER^2^S Intervention Feedback

#### 3.2.1. Community

Half of MCC physicians were willing to use the precision engagement tool (mean = 3.0) (Table 4). While the overall mean helpfulness score was 3.75, the highest rated functionality was to include a list of community physicians who practice in areas with high proportions of minority patients and their associated referral patterns to facilitate targeted events and outreach (mean = 4.4). While some MCC physicians viewed the precision engagement tool as irrelevant to their role, they shared that the tool would be helpful to their department or across MCC as a whole. Suggestions included making the precision engagement tool more user-friendly, assigning designated staff to utilize the tool, and incorporating data on prevalent cancers across different geographic areas.

##### Community Health Educators

All MCC patients and community residents believed that community health educators (CHEs) focused on CCT outreach and education would improve the patient experience. They felt CHEs could help connect them to social or financial resources (mean = 5.0 for both groups) and provide support during the new patient process (mean = 4.8 for both groups). Additional suggestions included on-site promotion of services, patient portal navigation, assisting with treatment logistics, and providing supportive care. MCC patients also expressed the need for language services and assistance with transferring international medical records to MCC (Table 5).

#### 3.2.2. Institutional

The portfolio profiler scored the highest for helpfulness among physicians and CRCs (mean = 4.0). The highest scoring functionality was to show the number of open MCC trials by department and by cancer characteristics (mean = 4.1). The recruitment dashboard had an overall helpfulness score of 3.62 among MCC physicians and CRCs, and the highest scoring functionality for both groups was to display demographic characteristics of all MCC patients relative to MCC trial enrollees (mean = 4.05). The eligibility criteria calculator was rated the lowest among MCC physicians (mean helpfulness score = 3.60), who described concerns with the tool’s practical value, noted limited time for modifying CCT protocols, and questioned whether this tool would impact CCT enrollment for patients who face other barriers to CCTs (e.g., access to care).

#### 3.2.3. Institutional and Interpersonal

The trial connect portal was rated highly for helpfulness (mean = 4.27) and willingness to use the tool (mean = 4.07) among community physicians, MCC physicians, and CRCs. The highest rated feature was the ability for community physicians to send electronic patient referrals to MCC (mean = 4.60). The option for community physicians to search for open trials at MCC and communicate with MCC physicians about referrals was also rated highly (mean = 4.57; mean = 4.53). Suggestions included adding information on distance to the treatment site, social services, live CCT enrollment data, patients’ awareness of their referral to MCC, and information on specific biomarker criteria. End users also suggested easy integration of this tool into their workflow, continuous updates on the CCTs, and coordination with new patient intake.

#### 3.2.4. Individual

The CHOICES DA was rated highly among MCC patients (mean overall helpfulness score = 4.53). The highest rated functionality was the ability to share self-reported barriers to receiving care with MCC clinical teams (mean = 4.9), followed by the option to send questions electronically before visits (mean = 4.6). A mean helpfulness score of 4.3 was given for (1) integrating CHOICES DA into the patient portal and (2) generating a list of CCT-related questions for patients to discuss with their clinical team. Additional suggestions included having in-depth information on trials, sharing patient testimonials, tracking patient interest in CCTs, finding ways to empower patients, communicating with other patients in the portal, and creating a forum where patients can ask questions anonymously.

## 4. Discussion

To our knowledge, this study is the first to holistically obtain insights from the community and cancer center end users on CCT knowledge, barriers, and needs in parallel with input on interventions to reduce barriers to minority patient CCT referral and enrollment. A unique approach to this study was utilizing the SEM to guide the interviews and group emerging themes, which confirmed the presence of barriers and needs to CCT referral and enrollment among patients, physicians, CRCs, and community residents in the same study, supporting previous findings [1,12,13,14,16,17,31,38,41,42,49,50,51]. Integrating quantitative measures to assess the utility of the ACT WONDER^2^S components validated the value of the proposed interventions in reducing barriers to minority CCT referral and enrollment. This comprehensive approach informed ACT WONDER^2^S to systematically address the target populations’ key needs.

### 4.1. Community Outreach and Engagement Strategies

End users expressed the need for community outreach and engagement strategies to promote CCT awareness and for CHEs to provide additional patient support, similar to previous reports [16,30,52,53,54,55]. Fostering mutual relationships and trust between cancer centers and the communities they serve is crucial in embodying community needs and priorities into clinical and translational research [30,56] and can mitigate factors precluding CCT participation across institutions and their geographic catchment areas [57]. Yet, most interventions focused on increasing CCT awareness, referral, and enrollment in underrepresented populations have targeted barriers at the cancer patient and physician levels, respectively [58]. Thus, seeking input from our end users confirmed the value of incorporating community outreach and education strategies to enhance CCT education, improve the patient experience, and provide supportive care (e.g., resources on available programs and support services at MCC) as a component of ACT WONDER^2^S.

### 4.2. Institutional and Interpersonal Approaches

Reported institutional-level CCT challenges included a lack of awareness and resources on available CCTs, delays in receiving external referrals, and inconsistent referral patterns. Previous reports observed similar challenges, including difficulties understanding referral processes [59] and inconsistent referral processes limiting CCT opportunities for minority populations [16,17]. Notably, our findings suggest these institutional barriers are interwoven with interpersonal experiences, including a lack of ongoing communication between physicians and inconsistent communication preferences across institutions. For instance, some MCC and community physicians in this study preferred direct communication across institutions about available CCTs and eligibility. Similar findings were observed in a study that found that referring providers will normally send patients to oncologists with whom they have previously collaborated [19]. While end users reacted positively to the ACT WONDER^2^S institutional-level digital tools, the trial connect portal was ranked the most positively overall, likely because this tool addresses the most common challenges end users described at both the institutional level (e.g., facilitation of CCT identification, rapid referrals) and interpersonal level (e.g., providing a digital platform for community and MCC physicians to easily communicate about referrals and trial availability). Eliciting feedback on barriers in conjunction with the ACT WONDER^2^S digital tools underscores the potential impact of this tool in alleviating complex CCT referral processes and facilitating more streamlined communication processes.

### 4.3. Patient-Level CCT Decision-Making

Individual-level barriers to CCTs are consistent with the literature, including cancer patients’ limited CCT knowledge, awareness, and hesitancy, which can ultimately lead to patients declining participation [3,4,5,6,7,8,9,10]. While previous interventions aimed to increase CCT awareness and knowledge for patients and providers, most studies relied on one or a few simple strategies, including patient navigation [60,61], decision support tools [23,24], or cultural/linguistic adaptations of single-session education sessions [28,61,62,63] to increase CCT knowledge. This study expands on previous work by engaging end users in refining digital and community interventions designed to increase CCT knowledge, awareness, and facilitate decision-making for minority patients, physicians, and community members. Collectively, all strategies were well received, adding utility to our MLI. Future studies seeking to evaluate MLIs should consider engaging end users early in the development process to facilitate the development of targeted interventions that address CCT knowledge barriers among relevant target populations.

### 4.4. Limitations and Strengths

This study is not without limitations. First, due to the small sample size, we recognize that our findings may not reflect some segments of the community and cancer center populations ACT WONDER^2^S aims to serve. While this study aimed to collect preliminary feedback on several topics, our sample size also limited the ability to detect statistically significant associations for our quantitative measures. ACT WONDER^2^S is also a single center study, and end-user perspectives may be limited to barriers and facilitators to CCTs at Moffitt Cancer Center, limiting the generalizability of our findings to other cancer center populations. Likewise, the landscape of CCTs continues to evolve, with more trials now including international sites [64,65], thus warranting future studies on how barriers and facilitators to CCTs may differ across regions. End users were also exclusively asked about ACT WONDER^2^S conceptually and were thus unable to provide personal experiences about using the digital tools or with community outreach and education directly. However, the qualitative interviews described in this study is the first of a two-phased approach for the ACT WONDER^2^S study development process; while not described here, phase 2 included additional qualitative interviews utilizing learner verification (LV) [66] and user-centered design (UCD) [67,68] approaches to finalize intervention components based on how they were refined from the feedback described in this study, which is a strength of our interview process.

## 5. Conclusions

In summary, findings reveal that community and cancer center populations face barriers to CCTs as posited within the SEM. Importantly, most end users were enthusiastic about leveraging community and digital strategies to reduce barriers to minority CCT referral and enrollment at the individual, interpersonal, institutional, and community levels. Our interviews confirmed the need for comprehensive approaches to address barriers to CCT referral and enrollment. This formative research approach is the basis for the ACT WONDER^2^S study that will be conducted across 14 geographic zones in the catchment area of our center through a cluster randomized design [69]. Our formative approach could serve as a model for developing future MLIs that aim to address further challenges to minority clinical trial referral and enrollment across levels of the SEM.

## Figures and Tables

**Table 1:** Overview of ACT WONDER^2^S.

**Community Outreach and Education Interventions**	**Description**	**End User**
Enhancing Patient Support	CHE patient support, including support in the new patient process, connecting patients to social or financial resources, relaying questions to clinical care teams, and answering questions about the new patient process via phone or email.	MCC patients, community residents
Clinical Trial Education	Clinical trial education sessions for the broader community.	Community residents
Continuing Medical Education	Education sessions on advances in cancer treatment, information on referral and enrollment into clinical trials, and implicit bias training.	Community physicians, MCC physicians
**Digital interventions**	**Description**	**End user**
Precision Engagement Tool	To identify geographic areas for Black/African American (AA) and Hispanic patient and physician cancer clinical trial (CCT) outreach.	MCC physicians
Portfolio Profiler	To view open trials at MCC and to explore potential gaps in the CCT portfolio relative to the cancer burden of MCC patients.	MCC physicians
Recruitment Dashboard	To display CCT enrollment rates and demographic characteristics compared to the overall MCC patient population.	MCC physicians, CRCs
Eligibility Criteria Calculator	To explore the impact of CCT criteria on patient eligibility.	MCC physicians
Trial Connect Portal	To facilitate rapid referral of patients to CCTs by (1) assisting with trial identification and (2) embedding communication between MCC clinical teams and physicians in the community.	MCC physicians, CRCs, community physicians
CHOICES Decision Aid (DA)	A self-guided interactive website to improve patient decision-making related to CCT participation.	MCC physicians

**Table 2:** Demographic characteristics of participants from the ACT WONDER^2^S interviews.

	MCC Participants			Community Participants	
	MCC Physicians (n = 10)	MCC CRCs (n = 10)	MCC Patients (n = 10)	Community Physicians (n = 10)	Community Residents (n = 10)
Age; M (SD) ^a^ range (in years)	43.3 (8.04) 36–62	28.4 (3.29) 22–34	57.8 (14.05) 29–79	45.7 (8.68) 33–58	55.8 (16.41) 25–70
Sex; n (%)					
Female	3 (30%)	5 (50%)	5 (50%)	5 (50%)	4 (40%)
Male	7 (70%)	5 (50%)	5 (50%)	5 (50%)	5 (50%)
Race; n (%) ^a^					
White	3 (30%)	4 (40%)	3 (30%)	5 (50%)	4 (40%)
Black or African American	2 (20%)	2 (20%)	5 (50%)	3 (30%)	5 (50%)
Asian	4 (40%)	2 (20%)	1 (10%)	1 (10%)	0 (0%)
More than one race	1 (10%)	2 (20%)	1 (10%)	1 (10%)	1 (10%)
Ethnicity; n (%) ^a^					
Non-Hispanic	9 (90%)	8 (80%)	5 (50%)	6 (60%)	6 (60%)
Hispanic	1 (10%)	2 (20%)	5 (50%)	4 (40%)	4 (40%)

^a^ Race and ethnicity data were collected separately from two different questions. For race: “Please select the group that best represents your race”; for ethnicity: “Do you consider yourself Hispanic or Latino”?

**Table 3:** Exemplary quotes from participants grouped by levels of the socioecological (SEM) model.

Themes	Exemplary Illustrative Quotes
**Community**	
Clinical trial education is needed for the broader community	“Even though Moffitt does, I think, do a decent job of physician liaisons and outreach for patient referrals in general, I don’t think we do nearly as good a job of disseminating these clinical trial ideas out to the community. And that sometimes has to be done in the form of conferences and things like that that are put on by the various departments, too…incorporating the clinical trial piece to the outreach process I think would help tremendously.” (MCC Physician 1047)
Education should happen prior to becoming a patient	“…so there’s the community education, and then there’s educating the specific patient. I think part of the problem is that you don’t have time to educate someone when they’re coming through the door, so that’s where the community piece is important.” (MCC Physician 1044)
Interest in public educational events	“…I’ve gone through with my churches. They have health fairs. We just had one October 7th were we had a variety of everything that you could think of, breast cancer. They had a gynecologist there. They talked about prostate cancer, cholesterol. They took people’s blood pressure, cholesterol, anything you could think of…And the churches in my community cater to that as well.” (MCC Patient 1042)
Building trust through community outreach	“Well, for many years that population have not really trusted the medical community for many reasons as you may know or may not…And I think that’s getting better as time goes on with the new generation. But at the same time, it’s not. The more people that have a better experience with it moving forward, then they can spread the word of why it’s important. So, just a historical thing that have occurred.” (Community Physician 1050).
A need to strengthen partnerships with community clinics	“Because I think oftentimes, whether we like it or not, these minority patients are being seen at places like the VA [Veterans Affairs] hospital. They’re county hospitals. So, oftentimes at those hospitals, the last thing on their provider’s minds is to send these patients in for a clinical trial because they’re probably not aware that the trial is ongoing. And really, they may think that it’s going to be impossible for those patients to get into Moffitt. So, again, I think it comes down to education and maybe even directed education at those sites. Although you are also going to have to look at the return on your investment.” (MCC Physician 1047)
Coalition building with other community organizations	“I believe that there was a better collaboration with the American Cancer Society liaisons in the past. I don’t even know if we have an ACS liaison in this area anymore. But we used to literally have people come around to the office and talk about, well, you know, Moffitt’s doing this or whatever it may be. And so I don’t know if Moffitt maybe has some community liaisons that would in-person kind of making that connection and get people engaged, but I think that there is some value in that.” (Community Physician 1013)
**Institutional**	
The importance of coordination, communication, and ongoing education for clinical trial coordinators	“…the treating physicians will contact either myself if they are within Moffitt, or they’ll contact the PI of the study if they are outside of Moffitt. So, they connect with one of our doctors at Moffitt…If the patient is already a Moffitt patient, it’s easier because all that happens is that a treating physician will reach out to me…So, they ask me to do my work, and then I go do my research on this patient, their history, their medical records, and match that to the study, and see if they are actually eligible or not eligible… **So, the work that has kind of a connection is** not easy, because there’s a web that you’re at the center of as a coordinator…What we try to do is to be trained on each other’s studies. Because you have to know that someone there is backing you up if you have an emergency, if you’re on PTO [paid time off], etc. So, this is something important.” (CRC 1012)
Trial awareness is limited across departments at MCC	“So, when I was at Moffitt, it’s such a big place, it’s got a lot of different departments. Very hard to keep up with what’s happening everywhere without talking to someone in that department… And then also, the clinical trials, coordinators a lot of times, there is a lot of turnover, even among the staff that run these things. So, ultimately a lot of times you kinda have to talk to the doctors in the department to know what’s going on research-wise there.” (Community Physician 1002).
External inquiry and referral processes	“…..most of the doctors that know about us are from specific hospitals or areas that do not have a lot of patients of color. We have to reach out to more areas that we know that these have majority of patients from Hispanic or from African American or from Middle Eastern or from Asian ethnicities…. Because I think the way our connection works is just with specific doctors that we know, or people at Moffitt. And we limit ourselves to the patients of those areas that mainly might not have a lot of people of color.” (CRC 1012)
Delays with external patient referrals can negatively impact enrollment	“And if we’re talking about Moffitt in particular this has become a bigger issue recently because there is a big process on getting an appointment which can be very slow. So, you tell a patient they have malignancy, and this is what you offer, and you offer them a second opinion. And then it takes three weeks or four weeks to even hear about an appointment. And sometimes the appointments are out a month and a half or something like that. Nobody wants to wait that long. I don’t want to wait that long.” (Community Physician 1051)
Insurance and financial barriers	“Okay, this is a big issue. If clinical trials are covered but our patients can’t get in until they get a referral with insurance that Moffitt takes, that’s another reason why they never get into clinical trials. Because then when I call the doctors and I say, “I have this patient with this disease I don’t know how to treat.” And then I say to them, “do you take the patient’s insurance,” they don’t know. They never know. The patients can’t get in there unless they are referred, even if they might be on a clinical trial.” (Community Physician 1037)
**Interpersonal**	
Trust and communication with providers	“Well, the reason I’m doing [the clinical trial participation] now is because of the doctor. I trust [name of doctor]. And when the screener called me and told me [name of doctor] recommended me, I trust him. So, I said, “Well, if he feels I’ll be a good candidate for it, I trust him enough that I would do this.” But normally, like I said, doing any type of trial situation, I usually don’t do it until I know a lot more about it. But that comfort and trust in him is why I’m doing this now. (MCC patient 1043)”
Informed decision-making	“Yeah. Given my history, I would want them to know if there’s any risks or any issue with me because of my cancer history doing this particular trial for this type of drug. I definitely would consult with them…So, I’m gonna read and do my due diligence before I step forward.” (MCC Patient 1043)
**Individual**	
Limited understanding and awareness of trials	“in movies or in TV shows people do clinical trials for extra money or things like that. (Community resident 1021)”
Hesitancies toward clinical trial participation	“They want people to join, so they know what this drug or whatever therapy they have will help, let’s say, age group, ethnicity…I’m a little wary of it because… Like at the meeting, I asked her if I had cancer, and I was directed to a clinical trial, could my using that or being in that program hurt me? And she said no. She said the doctors there would make sure that I was taken care of. And if anything went wrong, that they would take me off and steer me in the right direction. But for myself, the barrier was: What if this hurts me and makes me sicker?” (Community Resident 1040)
Patients decline trial participation due to feeling overwhelmed	“When I went in for my initial diagnosis for cancer, they asked if I wanted to participate in a trial. And I said no at that time…… that was the first time being approached…I just decided it was too soon. I was too much overwhelmed with the actual diagnosis of cancer and trying to absorb the treatment plan and all that. It was just not a good time for my mind to wrap around doing something like that.” (MCC Patient 1043)
Patient education on clinical trials needed	“I find that that can be a significant barrier for people sometimes, is there’s still a misconception and kind of negative perception about what clinical trials are and what it means to be on a trial. I still see people, when I mention a trial, who say, “I don’t wanna get a placebo.” And a majority of the trials I do are in the metastatic setting, and those patients wouldn’t be getting a placebo. Right? It’s not ethical for them to be able to be on a trial like that. So I have to kind of spend some time to backtrack and re-educate a lot of my patients.” (MCC Physician 1045)
A need for community physician education	“**I’m not familiar** because I’m a gynecologist. So, I don’t usually refer to medical oncology, it’s usually GYN oncology…Specifically not for a clinical trial, but I have referred them to other facilities for cancer treatment.” (Community Physician 1005)

**Table 4:** Participant feedback on the ACT WONDER^2^S digital tools.

Quantitative Feedback		Qualitative Feedback
**Community**		
**Precision Engagement Tool ^a^**		
**Mean helpfulness scores**		**Suggestions for improvement ^g^**
Overall helpfulness	3.75	Assign designated staff to use the tool. Include the ability to identify geographic locations of healthcare providers and their referral patterns. Ability to identify areas for outreach based on prevalent cancers.
Willingness to use the precision engagement tool	3.0	
Identify geographic areas for targeted community physician and patient outreach	4.1	
Access to a list of community physicians in priority zones and referral patterns	4.4	
Access to services provided by CHEs	3.5	
**Institutional**		
**Portfolio Profiler ^b^**		
**Mean helpfulness scores**		**Suggestions for improvement**
Overall helpfulness	4.0	Include information on trial phase and slot availability. Allow for smartphone access. Make sure clinical trial information is frequently updated. The ability to search for clinical trial priorities.
Show the number of trials open by department and cancer characteristics	4.1	
Show the % of patients with specific cancer characteristics in your department	4.0	
Show the % of patients eligible for a trial by race, ethnicity, and cancer type relative to catchment area	3.9	
**Recruitment Dashboard ^c^**		
**Mean helpfulness scores**		**Suggestions for improvement**
Overall helpfulness	3.62	Provide sufficient information without overwhelming providers. Track ineligible patients for trials and reasons to identify barriers. Include longitudinal enrollment trends. Compare existing enrollment numbers with enrollment goals of MCC. Include slot availability, number of times a trial has been open, number of trial enrollees, and screening trends.
Show demographic characteristics of MCC patients versus trial enrollees	4.05	
Show referral and enrollment rates by race and ethnicity	4.0	
Show patient referral and enrollment rates by geographic region (e.g., catchment area)	3.6	
Show patient enrollment rate by trial sponsor	2.85	
**Eligibility Criteria Calculator ^d^**		
**Mean helpfulness scores**		**Suggestions for improvement**
Overall helpfulness	3.6	Include data of how many more minority patients would be eligible if criteria changed. Ability to view impact of insurance criteria Include population-based information to craft inclusion criteria for protocols.
Willing to enter trial criteria	3.4	
Show the number and percent of eligible patients by race and ethnicity	3.7	
Output a score of generalizability of trial criteria	3.4	
Compare the proportions of Black Hispanic patients vs. non-Hispanic white that would be excluded based on criteria	3.6	
Alter the eligibility criteria to determine whether the change lessens the disparity	3.9	
**Trial Connect Portal ^e^**		
**Mean helpfulness scores**		**Suggestions for improvement**
Overall helpfulness	4.27	Include distance to treatment site and social services information. Assign designated staff to collect clinical documents. Have assistance for insurance-related barriers. Use codified language and phrasing for search functions and usability. Have an appealing design. Include point of contact for trials.
Willing to use the precision engagement tool	4.07	
Ability to search for open trials at Moffitt (by cancer characteristics)	4.57	
Ability for community physicians to send electronic patient referrals	4.6	
Ability for community physicians to communicate with MCC physicians about referrals	4.53	
Ability to assess patient eligibility	3.57	
**Individual**		
**CHOICES DA ^f^**		
**Mean helpfulness scores**		**Suggestions for improvement**
Overall helpfulness	4.53	Have in-depth information on trials and patient expectations. Share patient stories. Include a forum for anonymous questions. Being able to track patient interest in a clinical trial. Patient empowerment. Include patient support groups in the portal.
Integrate the tool into Moffitt patient registration	4.3	
Help to generate a list of questions related to clinical trial participation	4.3	
Send questions electronically before visit	4.6	
Share with care team the specific barriers when receiving care	4.9	

^a^ The precision engagement tool is designed to help identify geographic areas for Black/African American (AA) and Hispanic patient and physician cancer clinical trial (CCT) outreach. Respondents for these items include MCC physicians (N = 10). ^b^ The portfolio profiler allows clinicians to view open trials at MCC and to explore potential gaps to the CCT portfolio relative to the cancer burden of MCC patients. Respondents for these items include MCC physicians (n = 10). ^c^ The recruitment dashboard is designed to display CCT enrollment rates and demographic characteristics of CCT enrollees compared to the overall MCC patient population. Respondents for these items include MCC physicians (n = 10) and MCC CRCs (n = 10). ^d^ The eligibility criteria calculator allows users to assess the impact of eligibility criteria on the exclusion of minority patients into CCTs. Respondents for these items include MCC physicians (n = 10) ^e^ The trial connect portal is designed to facilitate rapid referral of patients to CCTs by (1) assisting with trial identification and (2) embedding communication between MCC clinical teams and physicians in the community. Respondents for these items include MCC physicians (n = 10), CRCs (n = 10), and community physicians (n = 10). ^f^ The CHOICES DA is a self-guided, interactive website focused on improving patient decision-making related to CCT participation. Respondents for these items include MCC patients (n = 10); ^g^ Bulleted text describes thematic findings pertaining to suggestions end users provided to help improve the digital tools during the interviews.

**Table 5:** MCC patient and community resident feedback on community health educators for additional patient support.

	Mean (M) Helpfulness Score		Suggestions for Additional Patient Support ^a^	
	MCC Patients	Community Residents	MCC Patients	Community Residents
Provide support to you during the new patient process	4.8	4.8	Community engagement prior to becoming a patient On-site promotion of services Integrate patient education into the patient portal	Assistance with educational resources Logistics of treatment Supportive care
Connect you to social or financial resources	5.0	5.0		
Relay your questions to your care team	4.8	4.3		
Answer questions about participating in clinical trials via phone or email	4.3	4.5		
Answer questions about the new patient portal process via phone or email	4.5	4.7		

^a^ Bulleted text describe thematic findings pertaining to suggestions end users provided on ways community health educators could provide additional patient support.

## Data Availability

The datasets presented in this article are not readily available to protect participant confidentiality and privacy. Requests to access the datasets should be directed to the corresponding author.

## Abbreviations

The following abbreviations are used in this manuscript:

CCTs	Clinical Trials
MLIs	Multilevel Interventions
MCC	Moffitt Cancer Center
CRCs	Clinical Research Coordinators
ACT	Advancing Clinical Trials: Working Through Outreach, Navigation, and Digitally
WONDER^2^S	Enabled Referral and Recruitment Strategies

## Appendix A

**Table A1:** MCC physician clinical characteristics (N = 10).

MCC Physician Clinical Characteristics	Statistic
Years at MCC; M (SD) range	8.8 (5.79) 3–23
Oncology specialty; n (%)	
Medical oncology	9 (90%)
Radiology	1 (10%)
Clinical Program; n (%)	
Blood and Marrow Transplant and Cellular Immunotherapy	3 (30%)
Breast Oncology	1 (10%)
Cutaneous Oncology	1 (10%)
Genitourinary Oncology	2 (20%)
Malignant Hematology	1 (10%)
Radiation Oncology	1 (10%)
Thoracic Oncology	1 (10%)
Track; n (%)	
Clinical investigator	4 (40%)
Physician educator	5 (50%)
Physician scientist	1 (10%)
Rank; n (%)	
Assistant member	1 (10%)
Associate member	9 (90%)

## Appendix B

**Table A2:** MCC CRC clinical characteristics (N = 10).

MCC CRC Clinical Characteristics	Statistic
Years at MCC; M (SD) range	1.4 (0.54) 1–2.5
Department; n (%)	
Malignant Hematology and Myeloid	3 (30%)
Bone Marrow Transplant	2 (20%)
Thoracic	2 (20%)
Head and Neck	2 (20%)
Phase 1 Clinical Trials	1 (10%)

## Appendix C

**Table A3:** Community physician clinical characteristics (N = 10).

Participant Characteristic	Statistic
Age; M (SD) range (in years)	45.7 (8.68) 33–58
Practice Type; n (%)	
Private practice	1 (20%)
Group practice	4 (40%)
Medical center	3 (30%)
Number of patients seen per month; M (SD) range	451 (576.78) 160–2000
Volume of cancer patients seen and/or diagnosed per month (nononcologists only, N = 6); M (SD) range	3.88 (3.59) 0.3–10
Health care system offers cancer clinical trials; n (%)	3 (30%)
Has led a therapeutic cancer trial in the past 5 years; n (%)	4 (40%)
Has referred a patient outside of their health system for cancer care; n (%)	9 (90%)
Has referred a patient to MCC for cancer care; n (%)	8 (80%)
Has referred a patient outside of their health system to a therapeutic cancer trial; n (%)	6 (60%)
Has referred a patient to MCC for a therapeutic clinical trial; n (%)	4 (40%)
Has attended a MCC physician CME activity; n (%)	6 (60%)
Has engaged with a member of the MCC physician liaison team; n (%)	5 (50%)
